# A Hebbian learning rule gives rise to mirror neurons and links them to control theoretic inverse models

**DOI:** 10.3389/fncir.2013.00106

**Published:** 2013-06-19

**Authors:** A. Hanuschkin, S. Ganguli, R. H. R. Hahnloser

**Affiliations:** ^1^Institute of Neuroinformatics, University of Zurich and ETH ZurichZurich, Switzerland; ^2^Neuroscience Center Zurich (ZNZ)Zurich, Switzerland; ^3^Department of Applied Physics, Stanford UniversityStanford, USA

**Keywords:** mirror neurons, inverse problem, linear models, songbird, sensory motor learning

## Abstract

Mirror neurons are neurons whose responses to the observation of a motor act resemble responses measured during production of that act. Computationally, mirror neurons have been viewed as evidence for the existence of internal inverse models. Such models, rooted within control theory, map-desired sensory targets onto the motor commands required to generate those targets. To jointly explore both the formation of mirrored responses and their functional contribution to inverse models, we develop a correlation-based theory of interactions between a sensory and a motor area. We show that a simple eligibility-weighted Hebbian learning rule, operating within a sensorimotor loop during motor explorations and stabilized by heterosynaptic competition, naturally gives rise to mirror neurons as well as control theoretic inverse models encoded in the synaptic weights from sensory to motor neurons. Crucially, we find that the correlational structure or stereotypy of the neural code underlying motor explorations determines the nature of the learned inverse model: random motor codes lead to causal inverses that map sensory activity patterns to their motor causes; such inverses are maximally useful, by allowing the imitation of arbitrary sensory target sequences. By contrast, stereotyped motor codes lead to less useful predictive inverses that map sensory activity to future motor actions. Our theory generalizes previous work on inverse models by showing that such models can be learned in a simple Hebbian framework without the need for error signals or backpropagation, and it makes new conceptual connections between the causal nature of inverse models, the statistical structure of motor variability, and the time-lag between sensory and motor responses of mirror neurons. Applied to bird song learning, our theory can account for puzzling aspects of the song system, including necessity of sensorimotor gating and selectivity of auditory responses to bird's own song (BOS) stimuli.

## Introduction

Complex vertebrate motor behaviors are generated by dedicated cortical circuits. The organization of these circuits and the plasticity rules that lead to their development and that guarantee their maintenance are functionally related to neural activity in single units and across larger populations (Gallese et al., 1996; Rizzolatti et al., 1996; Rizzolatti and Craighero, 2004; Harvey et al., 2012). For example, neural activity often strongly co-varies with motor behavior, allowing for estimation of detailed limb movement parameters from mere single-neuron recordings (Georgopoulos et al., 1986; Schwartz et al., 1988) and facilitating neural prosthesis (Santhanam et al., 2006; Ethier et al., 2012). However, in other cases, the amount of firing variability in single neurons can be dramatically dissociated from behavioral variability. For example, in songbirds, two distinct premotor areas are responsible for the generation of different aspects of the same vocal behavior. On the one hand, the cortical area HVC is involved in generating stereotyped adult song; lesions of HVC lead to degradation of typical adult song toward more unstructured subsong typical of very young birds (Nottebohm et al., 1976; Aronov et al., 2008). On the other hand, its counterpart, the lateral magnocellular nucleus of the anterior nidopallium (LMAN) in very young birds is involved in subsong production and in adults it is involved in the production of very subtle song variability that is barely noticeable to the human ear (Aronov et al., 2008). Lesions of LMAN in juveniles abolish song learning (Bottjer et al., 1984), and lesions in adults reduce the already small variability of adult undirected songs (the songs not direct toward another bird), manifest for example by reduced fluctuations of sound pitch (Kao et al., 2005; Stepanek and Doupe, 2010).

**Figure 1:**
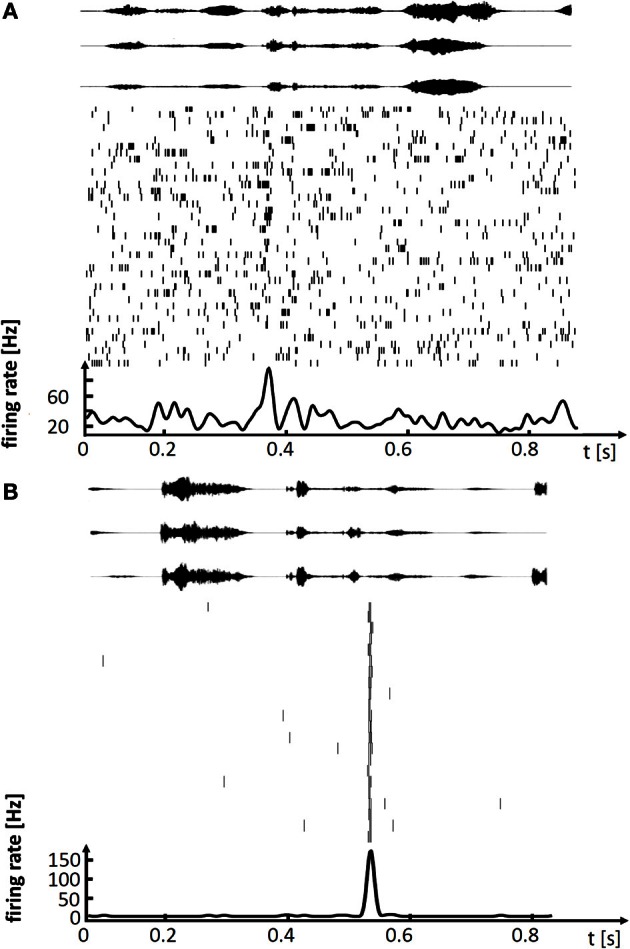
**Spiking activity in single neurons of singing zebra finches, illustrating (A) a variable premotor code in LMAN and (B) a stereotyped code in HVC. (A)** Spike raster plot of LMAN projection neuron aligned to 41 renditions of the stereotyped song motif. Three exemplary sound oscillograms of the motif are shown on top. The neuron produces single spikes and spike bursts at different times in each rendition of the motif. The motif-averaged firing rate is shown at the bottom. **(B)** Spike raster plot of HVC projection neuron aligned to 22 renditions of the stereotyped song motif (in a different bird), three exemplary motif oscillograms are shown on top. In each rendition of the motif the neuron produces a brief burst of spikes at precisely the same time.

These lesion studies ascribing differential roles of HVC and LMAN to song production, are paralleled by findings from electrophysiology. In HVC of singing birds, single principal neurons fire highly stereotyped spiking patterns associated with a given song syllable, with precision of individual action potentials in the sub millisecond range (Hahnloser et al., 2002; Kozhevnikov and Fee, 2007). By contrast, in LMAN of birds singing undirected songs, neurons fire very variable spike patterns, patterns that fluctuate on a trial-to-trial basis between loosely timed high-frequency bursts of action potentials and no spiking at all (Olveczky et al., 2005; Kao et al., 2008). Thus, stereotyped adult song is subserved by precise firing in HVC whereas subtle variability of adult song is subserved by large firing variability in LMAN, Figure 1. The diverse neural codes in LMAN and HVC are integrated in a dedicated nucleus that mediates both differential influences from these stereotypy and variability generators. Both HVC and LMAN project to the robust nucleus of the arcopallium (RA), which is the cortical output nucleus that directly innervates syringeal and respiratory motor neurons.

Whether stereotyped or variable, internal motor patterns responsible for generating behavior cannot be fully understood without considering the sensory input reaching the motor system. Indeed, the very development of motor systems as well as the formation of motor plans are profoundly shaped by sensory inputs. For example, the development of the mirror neuron system depends on sensorimotor experience (Catmur, 2012) and, the successful development of birdsong depends on intact HVC and LMAN activity during sensory exposure (Basham et al., 1996; Roberts et al., 2012).

We have learned much about the integration of sensory inputs into motor systems from single neuron studies examining responses during motor production and during matched sensory states. Among the key findings are mirror neurons that fire similarly when an animal executes a motor act and when it sees or hears another animal perform that same act. For example, mirror neurons in F5 of monkey premotor cortex fire both when the monkey touches an object and sees another subject touch that object (Rizzolatti et al., 1996; Rizzolatti and Craighero, 2004). Mirror neurons also exist in HVC of songbirds; these neurons fire at a precise time in the song, both when the bird sings the song and when it hears a similar song produced by another bird (Prather et al., 2008).

Mirror neurons establish a link between the observation of an act in another and self-generation of that same act. Such a remarkable correspondence between sensory and motor roles in single neurons has led to numerous suggestions about the function of mirror neurons in communication, imitation learning, cultural learning, and language development (Rizzolatti and Craighero, 2004; Oztop et al., 2012). Most importantly, mirrored responses have been proposed to be causally related to streams of motor and sensory activity (Oztop et al., 2006, 2012). A recent proposal is to tie properties of the mirror neuron system to correlative learning rules (Cooper et al., 2012). Accordingly, sensory responses in mirror neurons could develop from the contingency of motor-related firing and its sensory consequences feeding back to motor areas. Here we develop this idea and propose a simple mathematical theory of mirror neuron formation from correlational learning rules. To examine the critical role of motor variability, we study, based on earlier work (Hahnloser and Ganguli, 2013), mirror neuron formation for both motor codes with strongly correlated firing patterns among neurons, as in HVC, as well as for motor codes with uncorrelated firing patterns among neurons, as in LMAN.

We are particularly interested in relating mirror neuron properties to their computational role in control theoretic inverse models. Mirror neurons have previously been recognized as direct evidence of inverse models, which are models that transform desired sensory states into motor commands that can achieve those states and may be used for action generation (Oztop et al., 2012). From the control-theoretic perspective, internal inverse models give rise to mirrored responses because of the precise correspondence between a desired sensory target, the motor commands for producing that target, and the resulting sensory feedback. We pursue this idea and elucidate the conditions under which inverse models can arise from correlational learning during sensory feedback-dependent motor explorations.

We assume inverse models form in a context without prior knowledge of structure of either the motor apparatus or the delayed sensory feedback. We design an eligibility-weighted correlational learning rule that allows for the formation of both inverse models and mirror neurons. In the rule we propose, synaptic strengthening depends on contiguous co-activation of pre-and postsynaptic neurons, whereas synaptic weakening depends on heterosynaptic competition between sensory afferents innervating the same motor neuron. We argue that from a synaptic perspective, this rule is considerably simpler and more plausible than previously proposed rules and computational approaches toward systems-level inverse models based on error backpropagation (Jordan and Rumelhart, 1992). Our rule is most closely related to direct inverse model approaches (Miller, 1987; Slotine, 1987), in which, however, the possibility of unknown feedback delays has not been adequately addressed. Most importantly, we find that whether the formed mirror neuron system and inverse model is suitable for action imitation depends on the correlational structure of the neural code associated with motor production. Whereas a variable (explorative) motor code leads to causal inverse models and is suitable for mirror-neuron dependent action imitation, a stereotyped (repetitive) motor code leads to predictive inverse models and is not suitable for action imitation. Thus, our work provides an interesting link between the correlational structure of motor behavior, its underlying neural code, and fine-grained temporal properties of mirror neuron responses and their suitability for flexible action imitation.

Furthermore, these conceptual connections suggest a set of natural experiments designed to probe for the existence, and characterize the causal nature of, inverse models by measuring the fine grained temporal properties of the sensory and motor responses of mirror neurons. As we discuss below, when applied to the bird song system, these experiments make a specific, testable prediction about the existence and temporal properties of mirror neurons in the variable motor circuit LMAN, as well as explain the origin of previously observed temporal properties of mirror neurons in the stereotyped motor circuit HVC.

## Results

### A linear framework

**Figure 2:**
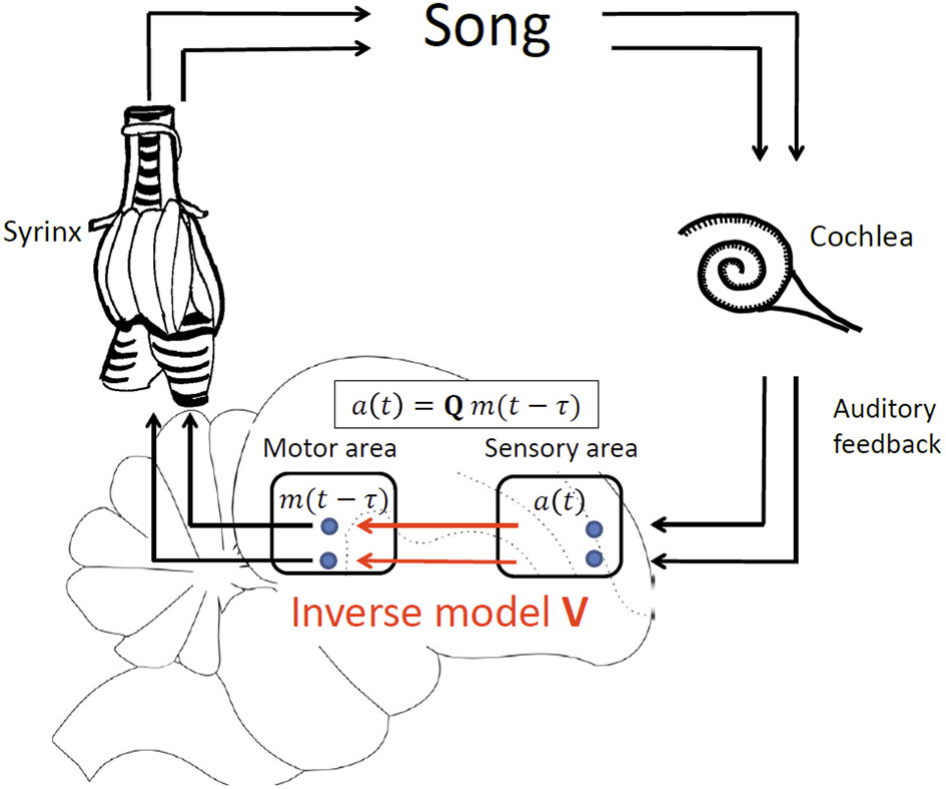
**Delayed feedback and inverse model, illustrated by vocal production in birds**. In our model of delayed sensory feedback the auditory response *a*(*t*) in a sensory area at time *t* depends linearly on motor activity *m*(*t* − τ) in a motor brain area at an earlier time *t*-τ according to *a*(*t*) = **Q***m*(*t* − τ), where **Q** is the unknown motor-sensory mapping and τ the unknown delay of auditory feedback. An inverse **V** is a mapping from sensory neurons back onto motor neurons that inverts the action of **Q**: **V** = **Q**^−1^.

We develop our theory in a simple linear framework in which the sensory response *a*(*t*) in a sensory brain area at time *t* is a vector of firing rates that is linearly related to the motor cause *m*(*t* − τ) at an earlier time *t* − τ, where *m*(*t* − τ) is a vector of firing rates in a motor area such as HVC or LMAN. The *time delay of sensory feedback τ = τ_*m*_* + τ_*a*_ is the sum of the time τ_*m*_ needed to translate motor activity into behavioral (vocal) output and the time τ_*a*_ it takes for a vocalization to elicit a sensory response. We assume a linear *motor-sensory mapping* modeled by the matrix **Q**, allowing us to specify the form of delayed sensory feedback as *a*(*t*) = **Q***m*(*t* − τ), Figure 2.

Note that for simplicity we assume linearity of the motor-sensory mapping **Q**. However, the simple linearity assumption inherent in **Q** need not be inconsistent with the existence of non-linearities between motor neuron activity and behavioral output (for example, song) and also with non-linearities between behavioral output and sensory responses. While it is the case that each of these transformations is highly non-linear, the dimensionality of motor behavior patterns realizable by muscle activity, or recorded by early sensory responses, is much smaller than the dimensionality of sensory or motor activity patterns deep within the cortex, by virtue of the fact that cortical motor and sensory neurons largely outnumber the few muscles and sensory receptors involved in the composite motor to sensory feedback loop. So for example, within the bird song system, it is thus probable that the low dimensional, composite non-linear transformation from cortical motor patterns, to muscle activity in the syrinx, to song, to cochlear response, back to cortical sensory feedback, could be well-approximated by a direct high dimensional linear map from the cortical motor area back to the cortical sensory area. This is in exact analogy to the theory of support vector regression approaches from machine learning, in which low dimensional non-linear maps can be well-approximated by high dimensional linear maps (Smola and Schölkopf, 2004). Thus, for our purposes, all we assume is that there exists at least one high dimensional linear map from cortical motor patterns to cortical sensory feedback patterns that approximates the composite feedback pathway implemented through the non-linear processes of motor generation and perception.

Now, an inverse model in this context is a mapping **V** = **Q**^−1^ expressed in the synaptic weights **V** from sensory onto motor neurons. Such a mapping allows sensory neurons to postdict the possible motor cause *m*^*a*^ of a sensory target (vector) *a* (either driven externally, recalled from memory, or resulting from a planning strategy) according to *m*^*a*^ = **V***a*. Such a postdiction ability of inverse models can be used in feedforward motor control in which the appropriate stream of motor commands *m*^*a*^(*t*) can be computed for a given desired sensory target sequence *a*(*t*) according to *m*^*a*^(*t*) = **V***a*(*t*).

The goals of our theory are to outline a biologically plausible, local mechanism for learning of the synaptic mapping **V** and to characterize the associated emergence of mirror neurons in this process.

### Eligibility-weighted Hebbian learning

**Figure 3:**
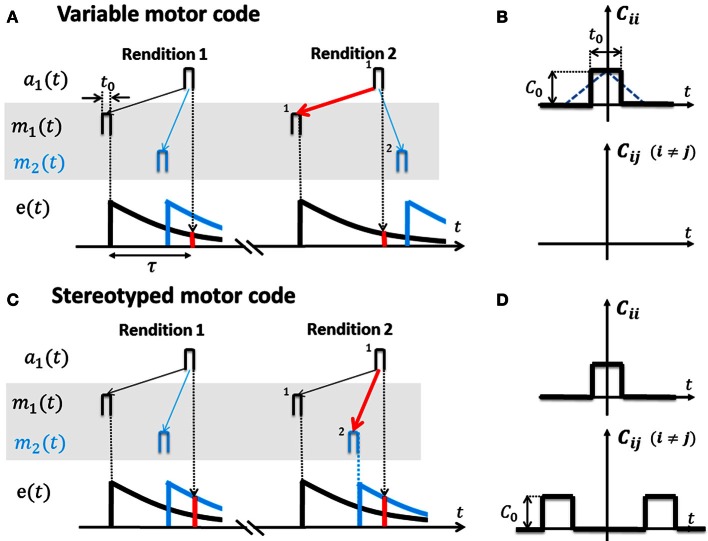
**Cross-correlation functions for variable and stereotyped motor codes. (A)** In a variable motor code *m*(*t*) (shaded area). Activity bursts *m*_1_ (black) and *m*_2_ (blue) of width *t*_0_ in two example motor neurons occur at diverse time lags relative to each other across renditions of the song motif. Auditory tuning in the shown sensory neuron is such that it responds *a*_1_ to bursts *m*_1_ after a time lag τ. Repeated co-activation *m*_1_ → *a*_1_ and non-zero eligibility *e*(τ) (red bar) at time lag τ leads to increased synaptic weight V_11_ (red arrow) and to a causal inverse. Lack of correlation between *m*_2_ and *a*_1_, as well as heterosynaptic competition, prevents V_21_ from similarly increasing (blue thin arrow). **(B)** The cross-correlation function *C*_*ij*_(*t*) for variable codes is flat except the auto-correlation peak at zero time lag (motor activity is uncorrelated among neuron pairs). Note: based on square activity pulses in motor neurons in **(A)** the true cross-correlation shape is triangular (blue dotted line) which we approximate by a square pulse of width *t*_0_ ≃ 10 ms. The auto-correlation peak height is *C*_0_. **(C)** In a stereotyped motor code *m*(*t*) (shaded area), bursts *m*_1_ (black) and *m*_2_ (blue) occur at a fixed time lag relative to each other across renditions of the song motif (traveling pulse of activity). Repeated co-activation *m*_2_ → *a*_1_ at higher eligibility (red bar) than the eligibility of *m*_1_ → *a*_1_ leads to strengthening of synapse V_21_ (red arrow) and to a predictive inverse. **(D)** The cross-correlation function *C*_*ij*_(*t*) for stereotyped codes peaks also at non-zero time lags.

We designed a simple learning rule in which potentiation of sensory-to-motor synaptic connections **V** arises from correlated firing in pairs of sensory and motor neurons. Because sensory feedback is delayed, synapses must be able to detect correlated firing within some non-zero time window, which we achieve by introducing an eligibility trace *e*(*s*) that establishes a link between activity at time *t* in a motor neuron and activity in a sensory neuron at a later time *t* + *s* (see also Figures 3A,C). The eligibility trace modulates the change in synaptic strength associated with correlated pre- and postsynaptic firing—it is a biophysical process that resides on the postsynaptic side of **V** synapses and is triggered by activity (i.e., spikes) in the postsynaptic (motor) neuron. Intuitively, we imagine that the spiking of a motor neuron, elicited for example from an internal source of motor variation that generates exploratory motor behavior, triggers the eligibility trace that in turn makes all synapses from sensory neurons onto that motor neuron eligible for future modification. Thus, if the delayed sensory feedback arrives to the sensory area within the window of eligibility, sensory to motor synapses can potentially learn to postdict the motor cause by correlating the current sensory feedback with past motor activity that might have generated it. We further assume that the eligibility is monotonically decaying in time, implying that sensory inputs preferentially connect onto motor neurons that were recently and reliably activated rather than motor neurons that were activated a long time ago. Necessarily, the decay of the eligibility trace must be slow enough to be able to attribute significant eligibility to sensory inputs with motor-to-sensory delays τ, which we subsume in the condition *e*(τ)» 0.

The full correlational learning rule describing changes in synaptic strength V_*ij*_ from auditory neuron *j* onto motor neuron *i* reads:

(1) $$\delta {\text{V}}_{ij}=\int_{0}^{\infty }\text{ds}\text{​}\left[e(s)m_{i}(t-s)a_{j}(t)\right]-{\hat{m}}_{i}(t)a_{j}(t),$$

where ${\hat{m}}_{i}(t)=\sum_{k}{\text{V}}_{ik}a_{k}(t)$ is the (silently) postdicted motor activity, corresponding to the summed auditory input to neuron *i* at time *t*. The subtractive term ${\hat{m}}_{i}a_{j}$ provides an equal time heterosynaptic depression (Lynch et al., 1977; Chistiakova and Volgushev, 2009) among all sensory afferent synapses onto a motor neuron. The strength of this depression depends on the amount of presynaptic activity but does not depend directly on postsynaptic activation. The utility of such depression is not only to stabilize activity but also to force synaptic connections towards inverse mappings as we will see. Note that we assume **V** synapses are “silently” correlating pre- and postsynaptic activity in Equation 1, i.e., **V** synapses do not contribute either to postsynaptic depolarization or to postsynaptic hyperpolarization. In other words, while the inverse model is being learned, the motor activity *m*_*i*_(*t*) is entirely driven by some other source than the afferent auditory input. Thus, from the perspective of extracellular physiology, it would appear that sensory feedback arriving to the motor area through the inverse model, is gated out of the motor area while that motor area is engaged in internally generated motor explorations.

In the following we examine the outcome of this learning rule in response to various forms of motor codes, with the goal of computing the synaptic weight matrix **V** at a steady-state of the learning rule, $\frac{\text{d}}{\text{dt}}\text{​}\langle V\rangle =0$, where 〈〉 denotes averaging over time (e.g., over different renditions of the song). To simplify the calculations, we assume motor codes with narrow spike-train cross-correlation functions, i.e., the width *t*_0_ of spike-train cross-correlation functions is much smaller than the characteristic decay time of the eligibility trace. Although such functions have not been extensively studied due to the difficulty of simultaneously recording from several neurons during singing, narrow cross-correlation is plausible for RA and HVC neurons because pseudo simultaneous recordings can be constructed from serial recordings thanks to high firing stereotypy in these cells, yielding cross-correlation widths on the order of 10 ms (Leonardo and Fee, 2005). Note that in LMAN, because of high firing variability, similar estimation of cross-correlation width is virtually impossible.

We model motor codes with diverse inherent levels of randomness. We model stereotyped motor codes by assuming that spike-train cross correlations extend over large time lags, in agreement with a traveling pulse of activity (Hahnloser et al., 2002; Harvey et al., 2012). We model variable motor codes by assuming that cross-correlations vanish except in a peak at zero time lag (white noise assumption), Figure 3B.

### A variable neural code yields causal inverses

If motor activity is uncorrelated among different neuron pairs, the resulting sensory to motor map **V** = *e*(τ)*t*_0_**Q**^−1^ equals the inverse of **Q** weighted by the eligibility at time lag τ (Equation A5, for the derivation see Appendix A3). Hence, **V** is a causal inverse that maps sensory representations onto their motor causes (in Figure 3A, auditory neurons map onto those motor neurons whose firing correlates most strongly with their own).

For example, during singing the motor cause *m*_1_(*t* − τ) (say a neuron that generates a 4 kHz tone) will frequently be followed by auditory response *a*_1_(*t*) (a 4 kHz detector neuron), leading to strengthening of synapse V_11_. By contrast, due to high variability of the motor code, associations between *m*_2_ (*t* − τ) (say a neuron that generates a 3 kHz tone) and *a*_1_ (*t*) are much less frequent (because the bird randomizes the production of 3 and 4 kHz tones). Hence, synapse V_21_ from the 4 kHz detector onto the 3 kHz generator will lose to synapse V_11_ due to heterosynaptic competition (Figure 3A).

### A stereotyped neural code yields predictive inverses

If the motor code is stereotyped and different motor neuron pairs are correlated at even very large time lags (extending over the full range of the eligibility trace and possibly beyond), then **V**≃*e*(0)*t*_0_**H**^τ^**Q**^−1^ is approximately a concatenation of the inverse of **Q** and a shifter matrix **H**^τ^ that maps motor activity at one time onto motor activity at a time lag τ later, i.e., **V** is a predictive inverse of **Q** (Equation A10). Under a predictive inverse **V**, a sensory neuron maps onto those motor neurons that were most recently active (and reliably follow in activation other motor neurons that give rise to the sensory neuron's response).

For example, during singing, the motor cause *m*_1_(*t* − τ) of a 4 kHz tone will frequently occur before the cause *m*_2_(*t*) of a 3 kHz tone (because the bird produces stereotyped downsweep syllables). Hence, the 4 kHz auditory detector response *a*_1_(*t*) will find much higher eligibility in motor neuron 2, leading to strengthening of V_21_ at the expense of V_11_, i.e., the 4 kHz detector neuron connects onto the 3 kHz generator neuron (Figure 3C).

### Lack of response to perturbed auditory feedback and selectivity for the BOS

During Hebbian learning of **V** in Equation 1 we required that synapses **V** are not able to drive spike responses in motor neurons during singing (**V** synapses learn silently). The main intuitive reason for the necessity of silent learning is that the learning goal of the inverse model synapses are to silently correlate the motor and sensory streams, without perturbing the motor stream that would result if sensory feedback were to pass through and drive spikes in the motor area. If the inverse model synapses allowed sensory feedback to significantly drive motor spikes, then the incoming sensory signals would serve to drive motor activity resulting in cyclic motor output with cycle time approximately equal to τ, i.e., birds would unavoidably produce repetitive motor output (stuttering).

Interestingly, there is much evidence for the gating out of sensory information in song motor nuclei. Principal motor neurons in LMAN and HVC do not respond to playback of white noise stimuli during singing (Leonardo, 2004; Kozhevnikov and Fee, 2007) and during states of high arousal (Cardin and Schmidt, 2003), though there are reports of distorted feedback responses in HVC interneurons in Bengalese finches (Sakata and Brainard, 2008). Lack of feedback sensitivity in principal motor neurons is usually ascribed to a form of gating caused by specific thalamic or neuromodulatory mechanisms (Dave et al., 1998; Schmidt and Konishi, 1998; Shea and Margoliash, 2003; Cardin and Schmidt, 2004; Coleman et al., 2007; Hahnloser et al., 2008), see also the Discussion.

By contrast, LMAN (Doupe and Konishi, 1991; Doupe, 1997; Solis and Doupe, 1999; Roy and Mooney, 2007) and HVC neurons (Katz and Gurney, 1981; Margoliash, 1983, 1986; Williams and Nottebohm, 1985) respond to auditory stimulation while birds are anesthetized or asleep, which we model as gating on of **V** synapses, i.e., we assume that auditory responses in motor neurons are driven via the learned inverse models.

The puzzling observation of the gating out of sensory inputs to motor areas during motor exploration, is naturally accounted for in our theory by necessity of correlating the current presynaptic sensory stream with past postsynaptic motor streams, to learn an unbiased inverse model (of unperturbed motor stream).

Also, interestingly, in both HVC and LMAN sensory responses are strongest for bird's own song (BOS) stimuli compared to other stimuli including the tutor song or the BOS played back in reverse time (McCasland and Konishi, 1981; Margoliash, 1986; Lewicki, 1996; Solis and Doupe, 1999). Such selectivity follows naturally from our model assumptions: for both stereotyped and variable motor codes, the mappings, whether causal or predictive, can only invert sensory responses that lie in the image of **Q** and cannot invert the full space of responses orthogonal to the image of **Q**. Such a restriction arises because only sensations that could arise through combinations of previously experienced sensory feedback during singing can actually be inverted into appropriate motor commands. In other words, the inverse model synapses map prior sensory feedback generated by the bird's own previous song into appropriate motor commands, but necessarily fails to map sensory activity patterns that are very different from the BOS into coherent motor patterns. Thus, assuming HVC and LMAN can be thought of as downstream of the output of an inverse model, our Hebbian learning rule generating inverse models can naturally account for the preference of sensory responses in HVC and LMAN for BOS; sounds very different from BOS are not appropriately inverted, and therefore presumably do not lead to coherent activation of motor patterns via sensory inputs propagating through the inverse model synapses.

### Inverse models and sensorimotor mirroring

The Hebbian learning rule in Section Eligibility-Weighted Hebbian Learning determines the wiring of sensory afferents into motor areas based on sensorimotor experience. How could one experimentally test for the existence of such wiring without painstaking, detailed inspection of anatomical connections and characterization of the sensorimotor mapping **Q**? Here we outline the design of experiments to probe for the existence of either causal or predictive inverse models. We propose to record from single neurons both in sensory and motor states and to compare motor activity and sensory-evoked responses using cross-correlation functions: as we will show, the time lag of peak cross correlation provides evidence for either predictive or causal inverses.

**Figure 4:**
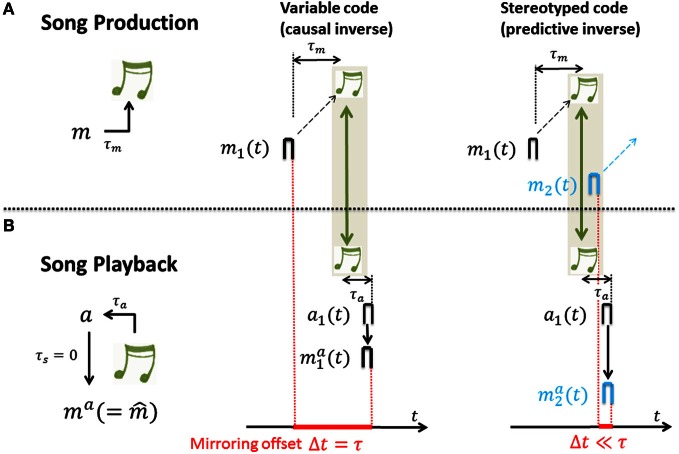
**The mirroring offset is determined in recordings of neural activity across (A) singing and (B) auditory stimulation by song playback; the offset (red) is large for causal inverses (middle column) and close to zero for predictive inverses (right column). (A)** During singing, a motor-neuron burst *m*_1_ drives a song feature (green notes) after a time delay τ_*m*_. **(B)** Playback of that song feature leads to auditory response *a*_1_ after a time delay τ_*a*_. And, auditory response *a*_1_ leads to motor neuron response *m*^*a*^_1_ in case of a causal inverse (middle column) and to motor neuron response *m*^*a*^_2_ in case of a predictive inverse (right column) after an additional time lag τ_*s*_ (spike propagation time from auditory to motor area) that is assumed to be 0 for simplicity. Thus, after alignment of motor activity and sensory response with song (green arrows), in the case of a causal inverse, the mirroring offset Δ*t* defined as the time lag between motor activity and playback response (red bar with extension set by red dashed lines) is equal to the sensory feedback delay τ = τ_*m*_ + τ_*a*_, whereas in the case of a predictive inverse the mirroring offset Δ*t* is close to 0. The reason for the 0 offset associated with a predictive inverse is that the auditory burst *a*_1_ driving the playback response *m*^*a*^_2_ is selective to the sound feature that during singing was generated by the much earlier burst *m*_1_ in a different neuron (black burst in **(A)**, right panel), but not the feature generated by *m*_2_ (blue burst in **(A)**, right panel).

In such mirroring experiments that we propose, a single neuron is first recorded during singing and then during playback of the just recorded songs while the bird is asleep in the dark (during which the auditory gate is open and motor neurons become responsive to auditory stimuli, presumably through an inverse model from an upstream sensory area). In our model, sensory responses $m_{i}^{a}(t)={\hat{m}}_{i}(t)=\sum_{k}{\text{V}}_{ij}a_{j}(t)$ during playback are driven via synaptic weights **V** (assumed to be at a steady-state of Equation 1, $\frac{\text{d}}{\text{dt}}\text{​}\langle V\rangle =0$). Computing the cross-correlation functions Corr(*s*) of the sensory response *m*^*a*^_*i*_(*t*) with motor activity *m*_*i*_(*t*) (as a function of time lag *s*) yields that (see Figure 4):

1. For variable neural codes we have that *m*^*a*^_*i*_(*t*) = *e*(τ)*t*_0_*m*_*i*_(*t* − τ) and the cross-correlation function Corr(*s*) is 0 except at time lags $s\in \left[\tau -\frac{t_{0}}{2},\tau +\frac{t_{0}}{2}\right]$ thus the peak correlation is near the sensorimotor time lag τ. In other words, for causal inverses the cross-correlation function between motor activity and sensory-evoked response peaks near time lag τ. That is, causal inverses are associated with large mirroring offsets equal to the loop delay τ. The reason is that the auditory response *m*^*a*^_1_(*t*) ∝ *m*_1_(*t* − τ) to song playback lags the song generating motor activity by a time lag τ. Intuitively, in a causal inverse model, if a motor neuron's activity is time locked to a particular song feature, it must fire before that feature in the motor production state, but after that feature in the sensory response state. This yields a temporal lag, or mirroring offset between the two (song-aligned) spike trains of a neuron recorded during motor production and during sensory exposure.
2. For stereotyped neural codes we have that *m*^*a*^_*i*_(*t*) ≃ *e*(0)*t*_0_*m_i_*(*t*) and the cross-correlation function Corr(*s*) as a function of the time lag *s* is proportional to the eligibility trace *e*(*s*). Thus, assuming a monotonic decay of eligibility, Corr(*s*) peaks at time lag *s* = 0. In other words, for predictive inverses the cross-correlation function peaks near zero time lag and predictive inverses are associated with close to zero mirroring offsets. The reason is that the auditory response *m*^*a*^_1_(*t*) ∝ *m*_1_(*t*) to song playback shows no lag with respect to the song generating motor activity. Intuitively, in a predictive inverse model, sensory feedback from a past motor action maps to concurrent motor activity in a stereotyped motor stream, which necessarily occurs after the motor activity that caused the sensory feedback. Thus, the past song elicits the firing of a motor neuron that generates future song. This implies that for any neuron there is no lag between its sensory and motor responses; the motor and sensory-evoked spikes of a motor neuron downstream of a predictive inverse model occur at the same time relative to song.

For derivations and model assumptions see Appendices A2–A4. In particular, here we assumed no synaptic delay between auditory and motor neuron, though this assumption can be relaxed. In summary, for both stereotyped and variable motor codes, sensory responses mirror motor activity. The amount of randomness in the motor code dictates the time lag of peak cross-correlation between motor activity and sensory-evoked responses, which we refer to as the *mirroring offset*. The mirroring offset thus serves as an important experimental observable that provides a window into fundamental differences in the types of inverse models that are computed by Hebbian learning, Figure 4.

Note that variable motor codes are associated with weaker mirroring than stereotyped codes, i.e., the cross-correlation functions for variable codes exhibit lower peak amplitudes than cross-correlation functions associated with stereotyped codes: In our model, the ratio of peak cross correlation is given by the eligibility at time lag τ divided by the eligibility at time lag zero (Equation A12 derived in the Appendices A3, A4). Thus, the steeper the eligibility trace, the weaker the mirrored response in case of variable motor codes. By contrast, the shape of the eligibility trace is expected to have almost no influence in case of stereotyped codes.

Note that the auditory response $m_{i}^{a}(t)=\sum_{k}{\text{V}}_{ik}a_{k}(t)$ in a motor neuron to song playback is mathematically identical to the (silently) postdicted motor activity ${\hat{m}}_{i}(t)=\sum_{k}{\text{V}}_{ik}a_{k}(t)$ defined after Equation 1, and used in learning the inverse model. Nevertheless, we use different symbols for these quantities to disentangle their meaning, i.e., the former being a superthreshold sensory response elicited in a quiet non-singing state of the bird, the latter being a subthreshold subtractive term that stabilizes synaptic learning during singing. The biophysical underpinnings of these two terms might largely be identical, with the silent nature of the posticted activity arising from some form of response gating (see also the Discussion).

### Gradient descent

We note that the learning rule in Equation 1 corresponds to gradient descent on the following error function:

(2) $$\text{E}(t)=\frac{1}{2}\sum_{i}\int_{0}^{\infty }{\left[m_{i}(t-s)-\sum_{k}{\text{V}}_{ik}a_{k}(t)\right]}^{2}\;e(s)\text{ds}$$

For a derivation, see Appendix A1. Thus, synaptic weights **V** converge such as to yield optimal postdiction ${\hat{m}}_{i}(t)=\sum_{k}{\text{V}}_{ik}a_{k}(t)$ of motor activity from sensory feedback. The origin of our eligibility-weighted Hebbian learning rule with heterosynaptic competition, from gradient descent of an energy function, confers a degree of robustness to the learning, as well as suggests generalizations to situations in which the synaptic transformation from sensory to motor areas is non-linear.

### Probabilistic models

More realistic neuron models are non-linear and contain spikes that are potentially probabilistic and certainly binary events. Also, more realistically, we may want to explicitly model intrinsic noise in motor and sensory-related responses rather than deal with motor variability only through their effects on cross correlations. As a first step to dealing with such realism, we have derived two probabilistic neuron models in which inverse models and mirroring can be studied in similar manners as in the linear model, outlined in the following.

In one of these models we calculate the influence of probabilistic (binary) responses on the strength of mirroring. We consider a random motor area that at any time can only be in one of two possible states *M* = 1 and *M* = 0 with prior probability $p(M=1)=\frac{1}{2}$. Assume analogously that the sensory area is such that a particular sensory feature is either detected (*S* = 1) or not detected (*S* = 0). We then model the relationship between motor activity and sensory consequence in terms of conditional dependencies between these two random variables. We assess the strength of mirroring in this model in terms of the cross-correlation coefficient between the two random variables (as derived in Appendix A5) and find the following result:

- For perfect auditory tuning (sensory neurons exhibit no noise to repeated sensory stimulation) the cross-correlation coefficient is given by the difference between the probabilities that the detected sensory feature is driven by the motor area vs. not driven by it. In other words, mirrored responses are proportional to the strength with which a single motor neuron contributes toward generation of the detected feature.
- In case of equal intrinsic noise in motor and sensory systems we find that the correlation coefficient is positive and proportional to the squared difference between the probabilities that the detected sensory feature is driven by the motor area vs. not driven by it.

Thus, the simple probabilistic model shows that the strength of mirroring may also be strongly reduced by the amount of intrinsic noise present in sensory and motor systems.

## Discussion

We have presented a simple model for the development of mirror neuron systems that is mathematically tractable, allowing us to relate mirror neuron properties such as the correlative strengths and the time lag of peak mirrored responses to the stereotypy (the correlation structure) of motor-related firing. Mirroring properties depend on the variability of the neural motor code which may be dissociated from apparent variability of the motor behavior as is the case in LMAN neurons that fire highly variable spike patterns despite high song stereotypy in adults. Our conclusions are valid for arbitrary sensory systems, provided they are able to signal sensory feedback from motor actions with sufficient sensitivity matched to the behavioral richness generated by the motor system (and of course provided that sensory afferents are subject to correlative Hebbian learning). In our derivation we have assumed that cross-correlation functions among motor neuron pairs are narrow, which was a simplifying assumption that allowed us to derive simple analytical forms of the sensory-to-motor mapping **V** and of mirroring properties. Approximate inverses should also result for motor codes with more complex time dependence, because by construction, the learning rule we considered corresponds to a gradient-descent rule that achieves minimal inversion error.

Although inverse models are attractive as models for vocal learning (Guenther et al., 2006; Hahnloser and Ganguli, 2013), they have previously been judged to be inappropriate for vocal learning in songbirds because of mainly two reasons: (1) young birds require many song repetitions with auditory feedback (Doya and Sejnowski, 2000), and (2) the learning schemes proposed either used a biologically implausible algorithm (Jordan and Rumelhart, 1992) or assumed the preexistence of an approximate inverse model (Kawato, 1990). Here we suggested a resolution to both of these issues and shown that in contrary to previous beliefs, inverse models constitute a potentially plausible framework for vocal learning in birds, too: the many song explorations used by young birds could be required to actually learn the high dimensional inverse model; and, the correlational learning we proposed is quite plausible and simple (but non-trivial nevertheless). This suggests potentially opening up the hypothesis space for learning rules operating within cortico-basal ganglia circuits, in both mammalian and bird song systems, to include models spanning the range from pure reinforcement learning (RL) to pure inverse model learning. Of particular interest would be intermediate learning rules that synergistically incorporate both dopamine-dependent plasticity thought to underlie RL as well, as Hebbian based plasticity shown here to mediate inverse model learning, in order to implement sophisticated model-based RL strategies. For example, a simple proposal would be that dopamine delivered to striatal synapses from the ventral tegmental area (VTA) might not be released purely nonspecifically, but instead might be delivered by an inverse model that can partially map errors in sensory coordinates to errors in motor coordinates, thereby guiding learning in ways more sophisticated than pure RL (O'Reilly and Frank, 2006).

The key to learning causal inverse models is motor variability. In motor areas such as HVC that fire stereotyped patterns, auditory afferents cannot disentangle cause-and-effect, leading to preferential formation of predictive inverses rather than causal ones. Predictive inverses have limited usefulness for action imitation from action observation, because under a predictive inverse, observation of a particular motor gesture will lead to imitation of the subsequent gesture in the imitator's motor repertoire, which may not be part of the actions to be imitated. For example, if a bird repeatedly sings *ABCD* during formation of the inverse and wants to later imitate repetitions of *ABDB*, then its predictive inverse will constrain it to produce repetitions of *BCDA* because perception of *A* maps to production of *B*, perception of *B* maps to production of *C*, etc.

Small temporal delays between motor activity and activity evoked by playback of BOS or BOS-resembling sounds have been reported previously. Prather et al. (2008) showed there is a small mirroring offsets of just a few milliseconds in HVC_X_ neurons of awake swamp sparrows and report similar (not quantified) results in Bengalese finches. Furthermore, Dave and Margoliash (2000) observed a small time lag of auditory-evoked activity also in RA neurons of sleeping zebra finches. Both these experimental findings reflect a predictive inverse. While predictive inverses have limited usefulness for action imitation they might provide stability in sequential vocalization. Indeed Sakata and Brainard report that perturbation of auditory feedback can change song syntax in Bengalese finches (Sakata and Brainard, 2006, 2008; Hanuschkin et al., 2011). By contrast, a causal inverse revealing itself by a large mirroring offset is maximally useful for song imitation. Indeed, preliminary results indicated a large non-zero mirroring offsets in LMAN (Giret et al., 2012).

An important element of our theory is the eligibility trace. To endow Hebbian learning with such a trace is necessary in realistic situations in which effects (sensory feedback) follow their cause (motor command) with some non-zero time lag arising from signal propagation delays, from twitch times of muscles, and from sensory and synaptic receptor latencies. In humans such a lag could span up to several hundreds of milliseconds, whereas in birds it may be as short as several tens of milliseconds. Eligibility traces also appear in RL theories (Seung, 2003; Fiete et al., 2007) and seem to be a general prerequisite for learning in the context of delayed feedback or delayed reward. We can imagine that neurons and synapses may hold decaying eligibility traces in terms of dedicated molecules such as calcium. Action potential generation is associated with rapid calcium entry that decays over the time course from several hundreds of milliseconds to a few seconds (McGeown et al., 1996; Wallace et al., 2008). The monotonic decay of intracellular calcium is well-suited to modeling a monotonically decaying eligibility trace. However, a monotonic decay of eligibility harbors both advantages and disadvantages. The disadvantage, as discussed, is the problem associated with stereotyped motor generators that can only hold predictive inverse models; to make inverse models causal, motor variability is required. Another way to guarantee causal inverse models—even under stereotyped motor explorations—would be to consider eligibility traces that do not monotonically decay but that peak at precisely the time delay inherent in closed sensorimotor feedback loops. The main caveat of such eligibility traces is that it may be questionable whether different muscles recruited for the same behavior must necessarily be associated with the same sensorimotor delay—and it is presently unclear how such variable delays could be matched to variable eligibility traces across synapses in a way that would ensure the learning of a causal inverse model. Moreover, phenomena such as speech co-articulation make it unlikely that there exists a constant sensorimotor delay across a large range of premotor neurons. The advantage, on the other hand, of a decaying eligibility trace is that sensorimotor contingencies and inverses can be learned regardless of sensorimotor latencies, providing robustness of sensorimotor learning.

Convergence of the sensory to motor synaptic weights toward inverses depends on details of the heterosynaptic competitive term. Heterosynaptic competitive terms have a certain appeal because of the useful normalization they provide (Fiete et al., 2010). In the context of this work, such terms imply locally available information at a single synapse about sensory inputs to other synapses. Though this information need not be provided instantaneously, we can only speculate about possible mechanisms for sharing such information among different synapses onto the same postsynaptic neuron. One possibility is that some form of intracellular signaling conveys this information. Another possibility to be explored is whether there exists an entire class of such competitive terms with a similar effect. For example, provided that motor and sensory codes are sufficiently sparse, it is conceivable that very simple subtractive terms might suffice for inverse formation. Whether other (even simpler) competitive terms result in approximate inverses needs to be further explored. We would like to point out preliminary evidence that inverses can be learned with Hebbian rules that include no heterosynaptic competitive terms (Senn and Pawelzik, pers. communication).

Our Hebbian learning theory has been analyzed so far in linear circuits, but we have indicated ways to overcome linearity by pinpointing extensions of our work to include nonlinear mappings and probabilistic neuron models. Further work will be required to test whether our correlative learning approach is suitable also for inverse model learning employing detailed biophysical models of the avian syrinx.

### Conflict of interest statement

The authors declare that the research was conducted in the absence of any commercial or financial relationships that could be construed as a potential conflict of interest.

## Appendices

### A1. Gradient descent derivation of eligibility-weighted hebbian learning

We can derive the learning rule in Equation 1 by gradient (steepest) descent on an error function E. The differential change δ**V** in synaptic weight is proportional to the gradient and we can write:

(A1) $$\delta V=-\frac{\text{dE}}{\text{d}V}.$$

The error function E_*i*_(*t*) for neuron *i* we define as the square difference between motor activity *m*_*i*_(*t* − *s*) and postdicted motor activity ${\hat{m}}_{i}(t)=\sum_{k}{\text{V}}_{ik}a_{k}(t)$, weighted by the eligibility associated with the time lag *s*.

$${\text{E}}_{i}(t)=\frac{1}{2}\int_{0}^{\infty }\text{ds }\text{​​​}{\left[m_{i}(t-s)-\sum_{k}{\text{V}}_{ik}a_{k}(t)\right]}^{2}\;e(s)$$

The total error is simply the sum of errors over all neurons E = ∑_*i*_*E*_*i*_.

By taking the gradient with respect to the *i*, *j* th weight only we find

$$\begin{array}{l} \frac{{\text{dE}}_{i}}{{\text{dV}}_{ij}}=-\int_{0}^{\infty }\text{ds​}\left[m_{i}(t-s)-\sum_{k}{\text{V}}_{ik}a_{k}(t)\right]\;e(s)a_{j}(t)\\ \;=-\int_{0}^{\infty }\text{ds​}\left[e(s)m_{i}(t-s)\;a_{j}(t)+\sum_{k}{\text{V}}_{ik}a_{k}(t)\;a_{j}(t)e(s)\right]. \end{array}$$

Assume a normalized eligibility trace (∫^∞^_0_*e*(*s*)ds = 1):

(A2) $$\begin{array}{l} \;\frac{{\text{dE}}_{i}}{{\text{dV}}_{ij}}=-\int_{0}^{\infty }\text{ds}e(s)m_{i}(t-s)\;a_{j}(t)+\sum_{k}{\text{V}}_{ik}\;a_{k}(t)\;a_{j}(t).\\ \Rightarrow \delta {\text{V}}_{ij}=\int_{0}^{\infty }\text{ds}e(s)m_{i}(t-s)\;a_{j}(t)-\sum_{k}{\text{V}}_{ik}a_{k}(t)\;a_{j}(t) \end{array}$$

#### A1.1. Extension to non-linear network

Note that our linear approach can be extended by introducing a nonlinear function *f* in the auditory to motor mapping in Equation 2:

$$\begin{array}{l} \text{E}=\frac{1}{2}\sum_{i}\int_{0}^{\infty }\text{ds}{\left[m_{i}(t-s)-f\left(\sum_{k}{\text{V}}_{ik}a_{k}(t)\right)\right]}^{2}e(s)\\ \;\Rightarrow \delta {\text{V}}_{ij}=\int_{0}^{\infty }\text{ds​}\left[m_{i}(t-s)-f_{i}\right]f_{i}^{'}a_{k}(t)e(s),\\ \;=\int_{0}^{\infty }\text{ds }m_{i}(t-s)f_{i}^{'}(t)\;a_{k}(t)e(s)-f_{i}(t)f{'}_{i}(t)\;a_{k}(t) \end{array}$$

Where *f*_i_ = *f*(∑_*k*_V_*ik*_ *a*_*k*_(*t*)) and *f*′_*i*_ = (∑_*k*_V_*ik*_*a*_*k*_(*t*))/dV_*ij*_.

#### A1.2. Probabilistic derivation of Hebbian learning rule

We derive a version of the Hebbian learning rule in Equation 1 that is based on the following probabilistic Boltzmann neuron model. For simplicity, we do not include the time dependence in the derivation (τ = 0). The auditory feedback response *a* given a motor activation *m* is given by the conditional probability

$${\text{P}}_{Q}(a|m)=\frac{e^{a^{T}Qm}}{Z_{Q}(m)},$$

parameterized by the matrix **Q**, which is the motor-sensory mapping (as before) and where

$$Z_{Q}(m)=\sum_{a}e^{a^{T}Qm}$$

is the partition function. The posterior probability of *m* is given by

$${\text{P}}_{Q}(m|a)=\frac{{\text{P}}_{Q}(a|m)\text{P}(m)}{\text{P}(a)}.$$

In a sensory state, auditory responses in motor neurons are driven via synapses **V** according to the probabilistic model:

$${\text{P}}_{V}(m|a)=\frac{e^{m^{T}Va}}{Z_{V}(a)}$$

with partition function

$$Z_{V}(a)=\sum_{m}e^{m^{T}Va}.$$

The error function in Equation 2 is replaced by the Kullbach-Leibler (KL) divergence between P_**Q**_(*m*|*a*) and P_**V**_(*m*|*a*):

(A3) $$\begin{array}{l} {\text{D}}_{\text{KL}}\left({\text{P}}_{Q}(m|a),{\text{P}}_{V}(m|a)\right)=\sum_{m}{\text{P}}_{Q}(m|a)\text{ln}\left(\frac{{\text{P}}_{Q}(m|a)}{{\text{P}}_{V}(m|a)}\right)\\ \;=\sum_{m}{\text{P}}_{Q}(m|a)[\text{ln}\left({\text{P}}_{Q}(m|a)\right)-\text{ ln}\left({\text{P}}_{V}(m|a)\right)]\\ \;=\sum_{m}{\text{P}}_{Q}(m|a)[\text{ln}\left({\text{P}}_{Q}(m|a)\right)+\;\ln\left(Z_{V}(a)\right)-m^{T}Va\;] \end{array}$$

Before taking the derivative of D_KL_ we compute the derivative of the partition function:

$$\begin{array}{l} \frac{\partial }{\partial {\text{V}}_{ij}}Z_{V}(a)=\sum_{m}e^{m^{T}Va}\frac{\partial }{\partial {\text{V}}_{ij}}m^{T}Va=\sum_{m}e^{m^{T}Va}m_{i}a_{j}\\ \;=Z_{V}(a)a_{j}\sum_{m}{\text{P}}_{V}(m|a)m_{i}\\ \;=Z_{V}(a)a_{j}{\langle m_{i}\text{|}a\rangle }_{m}, \end{array}$$

based on which it follows that

$$\frac{\partial }{\partial {\text{V}}_{ij}}\text{ln}(Z_{V}(a))=\frac{1}{Z_{V}\left(a\right)}\frac{\partial }{\partial {\text{V}}_{ij}}Z_{V}(a)=a_{\text{j}}{\langle m_{i}\text{|}a\rangle }_{m}.$$

Using this relationship and Equation A3 we can calculate the derivative of the KL-divergence with respect to V_*ij*_:

$$\begin{array}{l} \frac{\partial }{\partial {\text{V}}_{ij}}{\text{D}}_{\text{KL}}\text{​}\left({\text{P}}_{Q}(m|a),{\text{ P}}_{V}(m|a)\right)\\ \;=\frac{\partial }{\partial {\text{V}}_{ij}}\sum_{m}{\text{P}}_{Q}(m|a)\left[\text{ln}\left({\text{P}}_{Q}(m|a)\right)+\ln\left(Z_{V}(a)\right)-m^{T}Va\;\right]\\ \;=\sum_{m}{\text{P}}_{Q}(m|a)\left(\frac{\partial }{\partial {\text{V}}_{ij}}(\ln(Z_{V}(a)))-m_{i}a_{j}\right)\\ \;=\sum_{m}\frac{{\text{P}}_{Q}(a|m)\text{P}(m)}{\text{P}(a)}\left(\frac{\partial }{\partial {\text{V}}_{ij}}(\ln(Z_{V}(a)))-m_{i}a_{j}\right)\\ \;\Rightarrow {\langle -\frac{\partial }{\partial {\text{V}}_{ij}}{\text{D}}_{\text{KL}}\left({\text{P}}_{Q}(m|a),{\text{ P}}_{V}(m|a)\right)\rangle }_{a}\\ \;=\sum_{a,\;m}{\text{P}}_{Q}(a|m)\text{P}(m)\left(m_{i}a_{j}-\frac{\partial }{\partial {\text{V}}_{ij}}(\ln(Z_{V}(a)))\right) \end{array}$$

Thus, the gradient decent leads to

$$\Rightarrow \delta {\text{V}}_{ij}=m_{i}a_{j}-\frac{\partial }{\partial {\text{V}}_{ij}}(\ln(Z_{V}(a)))=\left[m_{i}-{\langle m_{i}\text{|}a\rangle }_{m}\right]a_{j}$$

This is the probabilistic analog of Equation A2, in which the silent postdictive motor activity ${\hat{m}}_{i}$ is replaced by the conditional expectation 〈*m*_*i*_*a*〉_*m*_ of activity in motor neuron *i* given the sensory response *a*.

### A2. Correlation of motor activity determines average synaptic change

The average synaptic change under learning rule Equation A2 satisfies

$$\begin{array}{l} \langle \delta {\text{V}}_{ij}\rangle =\int_{0}^{\infty }\langle m_{i}(t-s)a_{j}(t)\rangle e(s)\text{ds}-\langle \sum_{k}{\text{V}}_{ik}a_{k}(t)a_{j}(t)\rangle \\ \;=\int_{0}^{\infty }\langle \sum_{k}m_{i}\left(t^{\prime }\right){\text{Q}}_{jk}m_{k}\left(t^{\prime }+s-\tau \right)e(s)\rangle \text{ds}\\ \;-\;\langle \sum_{kml}{\text{V}}_{ik}{\text{Q}}_{kl}{\text{Q}}_{jm}m_{m}(t-\tau )m_{l}(t-\tau )\rangle . \end{array}$$

where we have substituted *t*′ = *t* − *s*. We can write this equation as

(A4) $$\langle \delta V\rangle =\left[\int_{0}^{\infty }e(s)C(s-\tau )\text{ds}-VQC(0)\right]Q^{T},$$

where C_*ij*_(*s*) = 〈*m*_*i*_(*t*)*m*_*j*_(*t* + *s*)〉 is the cross-correlation matrix of motor activity at time lag *s*. In the following we assume without loss of generality that the delay τ_*s*_ of synaptic transmission between auditory and motor neurons is negligibly small.

### A3. Variable motor code

#### A3.1. V is a causal inverse

We assume a motor code with a narrow correlation function that is non-zero only for small *t*_0_.

$$C(s)=\;\{\begin{array}{c} 1C_{0}\text{ for }\left|\text{s}\right|<t_{0}/2\\ 0\text{ otherwise} \end{array}$$

Where **1** is the unity matrix and *C*_0_ is a positive constant. The steady state solution 〈δ**V**〉 = 0 of Equation A4 leads to

$$\int_{0}^{\infty }\;e(s)C(s-\tau )\text{ds}-VQC(0)=0.$$

Assuming that the eligibility trace is constant over short time intervals of duration *t*_0_ (over which the correlation function is non-zero) yields

$$\Rightarrow e\left(\tau \right)t_{0}1C_{0}-VQ1C_{0}=0$$

$$\Leftrightarrow VQ=e(\tau )t_{0}1$$

This implies that the auditory to motor mapping **V** is proportional to the inverse of **Q** weighted by the eligibility at time lag τ:

(A5) $$V=e(\tau )t_{0}Q^{-1}$$

Thus, **V** is a causal inverse, at least when restricted to the image of **Q**.

#### A3.2. Variable motor codes are associated with large mirroring offsets

We simulate a mirroring experiment in which we cross correlate in a given neuron the motor activity *m*_*i*_(*t*) and the activity *m*^*a*^_*i*_(*t*) that results from observation of the motor act (achieved in birds by song playback though a loudspeaker).

The auditory response in motor neuron *i* is given by

$$\begin{array}{l} m_{i}^{a}(t)=\sum_{j}{\text{V}}_{ij}a_{j}(t)\\ \;=\sum_{j}{\left(VQ\right)}_{ij}m_{j}(t-\tau )\\ \;=e(\tau )t_{0}\sum_{j}\delta_{i,j}m_{j}(t-\tau ).\\ \;=e(\tau )t_{0}m_{i}(t-\tau ), \end{array}$$

Where *δ_i,j_* is the Kronecker-Delta, (*δ_i,j_* = 1 for *i* = *j* and *δ_i,j_* = 0 otherwise). Note that relative to song (either produced by the bird or played through the loudspeaker) the playback-evoked activity *m*^*a*^_*i*_(*t*) is shifted with respect to the motor activity *m*_*i*_(*t* − τ) by a time shift τ (as illustrated in Figure 4). The cross correlation Corr(*s*) between sensory-evoked and motor generated activity is defined as,

(A6) $$\text{Corr}(s)=\frac{1}{T'}\int_{0}^{T'}m_{i}(t)m_{i}^{a}(t+s)\text{dt}=\langle m_{i}(t)m_{i}^{a}(t+s)\rangle$$

Where *T*′ is the duration of the motor behavior (e.g., the song motif or song). Inserting the expression for the motor activity evoked by the auditory response into Equation A6 yields,

(A7) $$\begin{array}{l} \text{Corr}(s)=\langle m_{i}(t)e(\tau )t_{0}m_{i}(t-\tau +s)\rangle \\ \;=\{\begin{array}{ll} e(\tau )t_{0}C_{0} & \text{for}\left|\text{s}-\tau \right|<t_{0}/2\\ 0 & \text{otherwise} \end{array} \end{array}$$

Thus, the cross correlation is nonzero in a small time centered around τ. The peak cross-correlation value is given by the eligibility trace at time lag τ.

$$\text{CorrPeak}=t_{0}C_{0}e(\tau )$$

Note that our calculations are valid in principle for mean-subtracted *m*_*i*_(*t*). In case of non-mean subtracted *m*_*i*_ we should replace the cross correlation in Equation A6 by the cross covariance to obtain the same findings. However, in practice, mean subtraction is not necessary because the peak location (the mirroring offset) is independent of the mean.

### A4. Stereotyped motor codes

#### A4.1. V is a predictive inverse

We describe the motor activity by a traveling pulse $m_{i}(t)=\eta \text{​}\left(\frac{i}{\omega }-t\right)$ with speed ω, where

$$\eta (t)=\{\begin{array}{cc} 1 & \text{for}\left|t\right|<t_{0}/2\\ 0 & \text{otherwise} \end{array}$$

and *t*_0_ = 1/ω. The cross-correlation matrix for such a traveling pulse is a triangular pulse of height *t*_0_/*T* and width 2*t*_0_ which we approximate by a square pulse of width $t_{0},{\text{C}}_{ij}(s)≃\frac{t_{0}}{T}\eta \text{​}\left(\frac{i-j}{\omega }+s\right)$ in the following (illustrated in Figure 3D). To facilitate comparison of inverses associated with stereotyped and variable motor codes, we assume their peak correlations are identical, i.e.,

$$C_{0}=\frac{t_{0}}{T}.$$

At a steady state 〈δ**V**〉 = 0, Equation A4 yields

$$\int_{0}^{\infty }\;e\left(s\right)C(s-\tau )\text{ds}-VQC(0)=0.$$

Assuming again that the eligibility trace is constant over short time intervals of width *t*_0_, we find

$$\Rightarrow e\text{​}\left(\tau -\frac{i-j}{\omega }\right)t_{0}=VQ.$$

Given sensory input *a*_*j*_(*t*) and the inverse map **V**, the auditory-evoked activity *m*^*a*^_*j*_(*t*) is proportional to the eligibility trace:

(A8) $$\begin{array}{l} m_{i}^{a}(t)=\sum_{j}{\text{V}}_{ij}a_{j}(t)\\ \;=\sum_{j}{\left(VQ\right)}_{ij}m_{j}(t-\tau )\\ \;=e\left(t\text{-}\frac{i}{\omega }\right)t_{0}, \end{array}$$

defined for $t\geq \frac{i}{\omega }$.

By approximating the eligibility trace only by its maximum value $e\left(t-\frac{i}{\omega }\right)=e(0)\eta \left(t-\frac{i}{\omega }\right)$ we have that approximately

(A9) $$m_{i}^{a}(t)≃e(0)t_{0}m_{i}(t),$$

and so the playback-evoked activity *m*^*a*^_*i*_(*t*) is roughly identical to motor activity *m*_*i*_(*t*) (as illustrated in Figure 4).

To compute the matrix **V** we use the same approximation for the eligibility trace to obtain

$$\frac{1}{t_{0}}\text{​}{\left(VQ\right)}_{ij}≃e(0)\eta (i-j-\tau )=e(0)H_{ij}^{\tau },$$

where we have set ω = 1, and where **H** is a shifter matrix (also called cyclic permutation or circulant matrix), e.g., for *n* = 4:

$$H=\left(\begin{array}{cc} \begin{array}{cc} 0 & 1\\ 0 & 0 \end{array} & \begin{array}{cc} 0 & 0\\ 1 & 0 \end{array}\\ \begin{array}{cc} 0 & 0\\ 1 & 0 \end{array} & \begin{array}{cc} 0 & 1\\ 0 & 0 \end{array} \end{array}\right)\text{​}.$$

With this approximation we find that the synaptic mapping

(A10) $$V≃e(0)t_{0}H^{\tau }Q^{-1}$$

is the inverse of the motor map shifted in time by τ, i.e., **V** maps sensory activity evoked by motor activity at time *t* onto motor activity at time *t* + τ. In other words, the sensory lag is compensated and sensory-evoked motor activity at time *t* predicts motor activity at time *t*. Hence, **V** is a predictive inverse.

#### A4.2. Stereotyped motor codes are associated with small mirroring offsets

Based on the sensory-evoked activity *m*^*a*^_*i*_(*t*) derived in Equation A8 we find for the cross-correlation function Corr(*s*) between motor activity *m*_*i*_(*t*) and sensory-evoked activity *m*^*a*^_*i*_(*t*):

(A11) $$\begin{array}{l} \text{Corr}\left(s\right)=\frac{1}{T}\int_{0}^{T}m_{i}(t)m_{i}^{a}(t+s)\text{dt }\\ \;=\frac{t_{0}}{T}\int_{0}^{T}\eta \left(\frac{i}{\omega }-t\right)e\left(t-\frac{i}{\omega }+s\right)\text{​dt}\\ \;=\frac{t_{0}^{2}}{T}e(s)=t_{0}C_{0}e(s). \end{array}$$

Thus, the cross-correlation function is proportional to the eligibility trace. If the eligibility trace is monotonically decaying we find that the peak of Corr(*s*) occurs at *s* = 0 and is given by

$$\text{CorrPeak}=t_{0}C_{0}e(s).$$

In other words, stereotyped neural codes are associated with zero mirroring offsets.

The ratio of peak cross correlation for variable (A6) and stereotyped (A10) motor codes is given by

(A12) $$r=\frac{e(\tau )}{e(0)},$$

implying that the more stereotyped a neural code, the stronger is the observed mirroring effect.

### A5. Mirrored response strength in a probabilistic model

In the following we define a probabilistic model of a motor neuron that allows us to compute the mirroring strength, i.e., the correlation between motor activity and sensory-evoked activity. We assume a minimal model in which a neuron has only two states *R* = 1 (active) and *R* = 0 (inactive). In addition, we assume two behavioral states *B* = 1 (behavioral feature present), and *B* = 0 (behavioral feature absent). During motor production, the degeneracy of the motor code quantified by the conditional probability of neural activity given that the feature of interest is present during the behavior (e.g., the finger is extended or the song pitch is high) is

$$P_{M}(R=1|B=1)=p_{1}$$

and the probability that the neuron is active while the behavioral feature is absent (intrinsic noise) is

$$P_{M}(R=1|B=0)=p_{2}.$$

Hence, the average motor response [for prior *P*(*B* = 1) = 1/2] is given by

$${\langle R\rangle }_{\text{motor}}=\sum_{i}1\times P_{M}(R=1|B=i)P(B=i)=\frac{1}{2}\text{​}\left(p_{1}+p_{2}\right)=p.$$

In the sensory state (during observation of the behavior), the reliability of a response quantified by the conditional probability of triggering a sensory response given presence of the behavioral feature in the stimulus is given by,

$$P_{S}(R=1|B=1)=q_{1}$$

and the probability of a sensory response without the behavioral feature (intrinsic noise) is:

$$P_{S}(R=1|B=0)=q_{2}.$$

The parameters *p*_1_, *p*_2_, *q*_1_, and *q*_2_ can be freely chosen in this minimal model, for example *q*_2_ = *p*_2_ if intrinsic noise in sensory and motor states are assumed to be equal.

The average response in the sensory state is given by

$${\langle R\rangle }_{\text{sensory}}=\frac{1}{2}\left(q_{1}+q_{2}\right)=q.$$

The correlation between sensory and motor responses in this cell is

$$\begin{array}{l} \langle R_{\text{motor}}R_{\text{sensory}}\rangle =\sum_{i}1\times P_{M}(R=1|B=i)P_{S}(R=1|B=i)\\ \;\times \;P(B=i)=\frac{1}{2}\left(p_{1}q_{1}+p_{2}q_{2}\right). \end{array}$$

And, the correlation coefficient between motor- and sensory-evoked response is

$$\begin{array}{l} \text{CorrCoeff}=\frac{\langle R_{\text{motor}}R_{\text{sensory}}\rangle -\langle R_{\text{motor}}\rangle \langle R_{\text{sensory}}\rangle }{\sqrt{\langle R_{\text{sensory}}R_{\text{sensory}}\rangle \langle R_{\text{motor}}R_{\text{motor}}\rangle }}\\ \;=\frac{\frac{1}{2}\text{​}\left(p_{1}q_{1}+p_{2}q_{2}\right)-pq}{\sqrt{p(1-p)q(1-q)}}. \end{array}$$

We can discuss the following special cases:

- perfect sensory tuning (*q*_1_ = 1, *q*_2_ = 0, no instrinsic noise in sensory state):
  $$\text{CorrCoeff}=\frac{p_{1}-p_{2}}{2\sqrt{p(1-p)}}$$
- same tuning and same intrinsic noise in motor and in sensory states (*q*_1_ = *p*_1_, *q*_2_ = *p*_2_):
  $$\text{CorrCoeff}=\frac{{\left(p_{1}-p_{2}\right)}^{2}}{4p\left(1-p\right)}$$

In summary, the strength of mirrored responses scales linearly or quadratically with the contrastive probability that neural responses are locked to the behavioral feature vs. spontaneously driven.
